# Zusammenhänge zwischen psychischen Erkrankungen und Wohnungslosigkeit: Ergebnisse einer Sekundärdatenanalyse in einem Berliner Gesundheitszentrum für Obdachlose

**DOI:** 10.1007/s00103-022-03536-9

**Published:** 2022-05-04

**Authors:** Uwe Knörle, Stefan Gutwinski, Stefan N. Willich, Anne Berghöfer

**Affiliations:** 1 Gesundheitszentrum für Obdachlose der Jenny De la Torre-Stiftung, Berlin, Deutschland; 2 Versorgungsregion Wedding, Psychiatrische Universitätsklinik der Charité im St. Hedwig-Krankenhaus, Berlin, Deutschland; 3 Institut für Sozialmedizin, Epidemiologie und Gesundheitsökonomie, Charité – Universitätsmedizin Berlin, Luisenstr. 57, 10117 Berlin, Deutschland

**Keywords:** Psychische Erkrankungen, Wohnungslosigkeit, Obdachlosigkeit, Soziale Problemlagen, Inhaftierung, Mental disorders, Homeless persons, Homeless youth, Social problem, Imprisonment

## Abstract

**Hintergrund:**

Wohnungslosigkeit ist Ausdruck und Folge einer komplexen Problemlage, die die medizinischen und sozialen Versorgungssysteme in Deutschland vor große Herausforderungen stellt. Etwa 3 Viertel der wohnungslosen Menschen leiden an psychischen Erkrankungen. Ziel dieser Studie war es, Assoziationen zwischen psychischen Erkrankungen und Wohnungslosigkeit zu untersuchen.

**Material und Methoden:**

Es wurde eine Sekundärdatenanalyse von Patient*innendokumentationen eines Berliner Gesundheitszentrums für Obdachlose durchgeführt. In die explorative Studie eingeschlossen wurden Daten von 112 wohnungslosen Patient*innen, die dort zwischen den Jahren 2006 und 2020 versorgt wurden.

**Ergebnisse:**

Bei 84,9 % der Patient*innen lagen psychische Erkrankungen bereits vor dem Beginn der Wohnungslosigkeit vor. Assoziiert mit einem frühen Beginn der Wohnungslosigkeit waren die Faktoren niedrige Schulbildung sowie Drogenabusus. Eine lange Dauer der Wohnungslosigkeit war mit den Faktoren Alkoholabusus sowie Haftaufenthalte assoziiert. Jede erneute Episode der Straßenobdachlosigkeit war mit einer durchschnittlichen Verlängerung der Dauer der Wohnungslosigkeit um 7,9 Monate assoziiert.

**Diskussion:**

Da psychische Erkrankungen wichtige Einflussfaktoren für die Entstehung und Aufrechterhaltung von Wohnungslosigkeit sind, sollten vermehrt präventive Strategien sowie spezialisierte Angebote für diese vulnerable Gruppe geschaffen werden. Insbesondere wiederkehrende Episoden der Straßenobdachlosigkeit sollten so weit wie möglich verhindert werden. Der Zusammenhang zwischen Inhaftierungen und Wohnungslosigkeit legt nahe, dass eine intensivere Begleitung bei der Wiedereingliederung nach Haftentlassung erforderlich ist.

## Abstract (en)

**Background:**

Homelessness is an expression and consequence of a complex problem situation that poses major challenges to the medical and social care systems in Germany. About three quarters of homeless people suffer from mental illness. The aim of this study was to investigate associations between mental illness and homelessness.

**Material and methods:**

A secondary data analysis of patients’ records from a Berlin health center for homeless people was conducted. Data from 112 homeless patients who received care at the center between the years 2006 and 2020 were included in the explorative study.

**Results:**

Mental illness was present in 84.9% of patients before the onset of homelessness. The factors associated with an early onset of homelessness were low school education and drug abuse. A long duration of homelessness was associated with the factors alcohol abuse as well as imprisonment. Each new episode of street homelessness was associated with an average increase in the duration of homelessness by 7.9 months.

**Discussion:**

Since mental illnesses are important factors influencing the development and maintenance of homelessness, preventive strategies as well as specialized services for this vulnerable group need to be increasingly created. In particular, recurring episodes of street homelessness should be prevented as far as possible. The link between imprisonment and homelessness suggests that more intensive support is needed for reintegration after release from prison.

## Einleitung

Wohnungslosigkeit ist eine sozialmedizinisch komplexe und weiterhin ungelöste Problemlage in Deutschland. Trotz andauernder Bemühungen der Wohnungslosenhilfe steigt die Anzahl wohnungsloser Menschen nach Schätzungen der Bundesarbeitsgemeinschaft Wohnungslosenhilfe e. V. derzeit weiter an. Circa 41.000 Personen sollen dabei in Deutschland ohne jegliche Unterkunft in der Obdachlosigkeit leben, die eine extreme Unterform der Wohnungslosigkeit darstellt [1]. Obwohl bereits vorhandene Hilfsstrukturen stetig weiterentwickelt werden und die Kosten hierfür in Berlin beispielswiese jährlich steigen [2], kann insbesondere die gefährdete Gruppe der psychisch erkrankten wohnungslosen Personen dennoch oftmals nicht erreicht werden [3–5].

Inwiefern sich psychische Erkrankungen und Wohnungslosigkeit gegenseitig beeinflussen, ist weiterhin Gegenstand wissenschaftlicher Diskussion. Unstrittig ist, dass unter wohnungslosen Menschen im Vergleich zur Allgemeinbevölkerung erhöhte Prävalenzen psychischer Erkrankungen vorliegen: Einer aktuellen deutschen Metaanalyse zufolge leiden 77,5 % der wohnungslosen Frauen und Männer an psychischen Erkrankungen, die Rate zeigte sich erkrankungsübergreifend um das 3,8-Fache erhöht. Substanzbezogene Störungen machten dabei mit 60,9 % die häufigsten psychischen Erkrankungen aus, am zweithäufigsten wurden Angststörungen mit 17,6 % festgesellt, gefolgt von affektiven Störungen mit 15,2 % und psychotischen Erkrankungen mit 8,3 % [6]. Eine umfangreiche Metaanalyse der Universität Oxford, die Kohorten mehrerer westlicher Länder einschließt, kommt zu einer vergleichbaren Rangfolge. Die höchsten Prävalenzen psychischer Erkrankungen zeigten in dieser Studie die Alkoholabhängigkeit mit 8,1–58,5 %, die Drogenabhängigkeit mit 4,5–54,2 % und die psychotischen Erkrankungen mit 2,8–42,3 % [7]. Die erhebliche Streuung der Prävalenzen wird z. T. auf die Zusammensetzung und Größe der Studienpopulationen, die Studienregion und die vorhandene Versorgungsstruktur zurückgeführt. Jedoch erwiesen sich auch Faktoren wie die Teilnahmerate der wohnungslosen Menschen und die klinische Erfahrung der Untersucher als relevant. Höhere Prävalenzen von Alkoholabhängigkeit fanden sich in jüngeren Studien.

Über die Entwicklung psychischer Erkrankungen im zeitlichen Zusammenhang mit dem Beginn der Wohnungslosigkeit liegt weit weniger Evidenz vor. Steht zu Beginn eher die psychische Erkrankung oder die Wohnungslosigkeit? Verschiedene Studien zeigen zwar, dass psychische Erkrankungen bedeutende Risikofaktoren für einen möglichen Verlust der Wohnung sein können [3, 8]. So fand die SEEWOLF-Studie bei 85 % der Studienpopulation eine psychische Erkrankung vor Beginn der Wohnungslosigkeit [9]. Jedoch wurden die Teilnehmer*innen überwiegend aus psychiatrischen Versorgungseinrichtungen rekrutiert, was die Gefahr eines Selektionsbias beinhaltet. Im Gegenzug konnten die mit Wohnungslosigkeit verbundenen Lebensbedingungen und Stigmatisierungserfahrungen aber auch als Stressoren identifiziert werden, die das Auftreten einer psychischen Erkrankung beschleunigen oder einen ungünstigen Verlauf bewirken können [10].

Ferner gibt es Faktoren, die sowohl mit der Entstehung von Wohnungslosigkeit als auch mit der Entstehung psychischer Erkrankungen assoziiert sind. So werden beispielsweise belastende Lebensereignisse in der Kindheit und Jugend in der aktuellen internationalen Forschung zunehmend als Risikofaktor für Wohnungslosigkeit evaluiert [11–13], in Deutschland gibt es hierzu aktuell keine Studien. Wenig Berücksichtigung fand in der deutschen Wohnungslosenforschung zudem die Thematik der Straffälligkeit, die mit Entstehung und Aufrechterhaltung von Wohnungslosigkeit und psychischen Erkrankungen assoziiert zu sein scheint [14–16].

Detailliertere, insbesondere für deutsche Versorgungsverhältnisse geltende wissenschaftliche Erkenntnisse über die Zusammenhänge von psychischen Erkrankungen und Wohnungslosigkeit könnten für die Anpassung bestehender Hilfsangebote enorm hilfreich sein. Vor diesem Hintergrund wurden in der Studie folgende Hauptfragestellungen explorativ bearbeitet:

- Welcher zeitliche Zusammenhang besteht zwischen den psychischen Erkrankungen und der Wohnungslosigkeit? Wie verändern sich Anzahl und Schwere der psychischen Erkrankungen im Laufe der Wohnungslosigkeit?
- Lassen sich Faktoren identifizieren, die mit einem frühen Beginn der Wohnungslosigkeit assoziiert sind?
- Lassen sich Faktoren identifizieren, die mit einem langen Verlauf der Wohnungslosigkeit assoziiert sind?

## Methoden

### Studiendesign

Es handelt sich bei der vorliegenden Studie um eine Sekundärdatenanalyse auf Basis von Patient*innendokumentationen einer Population wohnungsloser Personen, die im Gesundheitszentrum für Obdachlose der Jenny De la Torre-Stiftung in Berlin in den Jahren 2006 bis 2020 betreut wurden. Das Zentrum ist ein niedrigschwelliges Angebot, das für Patient*innen neben einer medizinischen und psychologischen Betreuung auch Beratungen durch Rechtsanwält*innen und Sozialarbeiter*innen anbietet. Die Ausarbeitung wurde anhand der 2. Version der standardisierten Berichtsroutine für Sekundärdatenanalysen (STROSA) durchgeführt [17].

### Primärdatenquellen

Die der Studie zugrunde liegenden Primärdaten wurden von der Gründung des Gesundheitszentrums im September 2006 bis zum Beginn der Datenerhebung im Mai 2020 in ca. 3500 Patient*innendokumentationen in Papierform als Verlaufsakten dokumentiert. Die Routinedokumentation lässt sich in 5 Teile gliedern:

1. der standardisierte Anamnesebogen, der bei Erstkontakt vom medizinischen Fachpersonal erhoben wird,
2. die medizinische Verlaufsakte, die alle medizinischen Beratungsanlässe, die jeweilige Wohnsituation, diagnostische Maßnahmen, Diagnosen sowie Therapien durch behandelnde Fachärzt*innen dokumentiert,
3. externe medizinische Dokumente, die von anderen Einrichtungen zugeschickt oder von Patient*innen mitgebracht werden,
4. von der Psychologin geführte und dokumentierte klinische Interviews in Fließtextform. Hier finden sich eine biografische Anamnese, die Beschreibung der aktuellen psychologischen Situation sowie von ihr gestellte Diagnosen. Die Psychologin ist approbierte Psychotherapeutin und verfügt über umfangreiche Erfahrung in der Beurteilung psychisch Erkrankter, erworben im sozialpsychiatrischen Dienst, im Krisendienst, in verschiedenen Beratungsstellen für suchtmittelabhängige Personen sowie als Gutachterin im Gesundheitsamt,
5. Dokumente, die im Zuge der sozialen Beratungsanlässe erhoben werden. Zusätzlich Dokumente anderer Einrichtungen und staatlicher Institutionen (u. a. Gerichtsbeschlüsse, Schreiben der Jobcenter, Kostenübernahmen für Wohnheimplätze), die für die Sozialberatung relevant sind.

Aus den Dokumentationsteilen 1., 2. und 4. wurden Informationen zu Beginn, Länge und Ursachen der Wohnungslosigkeit entnommen.

### Auswahlkriterien

Eingeschlossen in die Studie wurden die Dokumentationen aller Patient*innen, die folgende Merkmale erfüllten:

- Volljährigkeit,
- zum Zeitpunkt des Erstkontakts im Gesundheitszentrum wohnungslos, gemäß ETHOS(Europäischen Typologie für Obdachlosigkeit, Wohnungslosigkeit und prekäre Wohnversorgung)-Kategorien: obdachlos (1–2), wohnungslos (3–7, umfasst alle zeitlich begrenzten Unterbringungen), ungesichert (8–10, umfasst temporäres Wohnen bei anderen Personen und drohende Zwangsräumung) und ungenügend (11–13, umfasst z. B. Wohnwägen oder Abbruchgebäude; [18]),
- Patient*innenkontakt mit Dokumentation an mindestens 2 Zeitpunkten,
- Vorhandensein eines möglichst vollständigen medizinischen Anamnesebogens sowie mindestens eines dokumentierten psychologischen Interviews.

### Statistische Methoden

Durch die genaue zeitliche Erfassung des Beginns der Wohnungslosigkeit, der im medizinischen Anamnesebogen dokumentiert wurde, konnte valide eingeordnet werden, ob Betroffene vor oder nach Eintritt der Wohnungslosigkeit erstmals psychisch erkrankt waren. Eine genaue Identifizierung, zu welchem Zeitpunkt welche psychische Erkrankung in der Biografie der Patient*innen begann, war nicht möglich, deshalb wurde mit kumulativen Inzidenzen in der gesamten Periode der Wohnungslosigkeit gearbeitet. Wiederholte Episoden der Obdachlosigkeit wurden definiert als Wechsel aus einer gesicherten Wohnform, entspricht den ETHOS-Kategorien 3–13 [18] sowie einer mietvertraglich abgesicherten Wohnform, zurück auf die Straße. Die Beschreibung der Studienpopulation durch soziodemografische und medizinische Merkmale erfolgte deskriptiv. Gültige Angaben zur Anzahl *n* sowie mögliche Mehrfachantworten sind vermerkt, auf die Imputation fehlender Werte wurde verzichtet. Die Fragestellungen wurden mittels zweiseitigen und ungerichteten t‑Tests, einfaktorieller ANOVAs (Post-hoc-Test: Games-Howell) und linearer Regression untersucht. Die Hypothesen wurden auf explorativem Niveau getestet, daher wurde auf eine Alphafehlerkumulierung verzichtet. Das Signifikanzniveau wurde auf einen Wert von 0,05 festgelegt. Die angegebenen Konfidenzintervalle (KI) beruhen auf Bootstrap-Ergebnissen (jeweils 1000 Bootstrap-Stichproben). Die statistischen Berechnungen erfolgten mit der Software IBM SPSS, Version 26 (IBM, Ehningen, Deutschland) [19].

## Ergebnisse

Im Gesundheitszentrum wurden ca. 3500 Patient*innen dokumentiert. Davon hatten 212 ein psychologisches Interview, welches die Dokumentation möglicher psychischer Symptome oder Störungen ermöglichte. Diese Patient*innen waren alle mindestens zweimal im Gesundheitszentrum vorstellig geworden. Hiervon wurden nach Anwendung der Ausschlusskriterien 100 Patient*innen ausgeschlossen (keine Wohnungslosigkeit: 10, nicht volljährig: 1, kein oder unvollständiger medizinischer Anamnesebogen vorliegend: 89). Letztendlich wurden die Dokumentationen von 112 Patient*innen in die Studie eingeschlossen.

### Soziodemografische Merkmale

Der überwiegende Teil der Patient*innen war männlich, knapp die Hälfte der Patient*innen hatte eigene Kinder, fast alle waren ohne Partnerschaft (Tab. 1). Dreiviertel der Patient*innen waren obdachlos. Die Dauer von Beginn der Wohnungslosigkeit bis zur Erstvorstellung im Gesundheitszentrum betrug im Median 2,0 Jahre.

**Table 1:** 

	M (SD)
*Alter, n* *=* *112*	43,1 (11,3)
*Alter bei Beginn der Wohnungslosigkeit, n* *=* *105*	39,7 (11,9) Spannweite 14–64 Jahre
*Dauer der Wohnungslosigkeit, n* *=* *105*	2,0 (3,6) Spannweite 2 Tage–16 Jahre
	*Anzahl (%)*
*Geschlecht, n* *=* *112*	
Männlich	88 (78,6)
Weiblich	24 (21,4)
*Familienstand, n* *=* *98*	
Ledig	65 (66,3)
Geschieden oder getrennt lebend	28 (28,6)
Verwitwet	3 (3,1)
Verheiratet	2 (2,0)
Eigene Kinder	47 (48,5)
*Wohnsituation*^a^*, n* *=* *112*	
Obdachlos	85 (75,9)
Davon auf der Straße lebend (Kat. 1)	40 (35,7)
Davon in Notunterkunft lebend (Kat. 2)	45 (40,2)
Wohnungslos (Kat. 3–7)	11 (9,8)
Ungesichert (Kat. 8–10) oder ungenügend wohnhaft (Kat. 11–13)	16 (14,3)
Kürzer als 1 Jahr wohnungslos	45 (42,8)
Länger als 1 Jahr wohnungslos	60 (57,2)
*Nationalität, n* *=* *112*	
Deutsch	107 (95,5)
Andere	5 (4,5)
*Schulbildung, n* *=* *100*	
Abitur	15 (15,0)
Realschulabschluss	36 (36,0)
Hauptschulabschluss	34 (34,0)
Ohne Schulabschluss	15 (15,0)
*Vorhandene Krankenversicherung, n* *=* *108*	60 (55,6)
*Einkommen, n* *=* *109 (Mehrfachantworten möglich)*	
Sozialversicherungspflichtige Beschäftigung	2 (1,8)
Sozialversicherungsfreie Beschäftigungen	44 (40,4)
Bezug von staatlichen Leistungen	55 (50,4)
Davon Arbeitslosengeld I	2 (1,8)
Davon Arbeitslosengeld II	37 (33,9)
Davon Erwerbsunfähigkeitsrente	12 (11,0)
Davon Altersrente	4 (3,7)
Ohne Einkommen	19 (17,4)
*Somatisch vorerkrankt, n* *=* *91*	61 (67,0)
*Raucherstatus, n* *=* *93*	67 (72,0)

*M* Mittelwert, *SD* Standardabweichung

^a^Kategorien nach ETHOS-Typologie

Bei 109 der 112 Patient*innen konnten die verschiedenen Einkommensquellen eruiert werden. Nur 2 Patient*innen hatten eine reguläre sozialversicherungspflichtige Arbeit. Etwa die Hälfte bezog staatliche Leistungen wie Arbeitslosengeld oder Renten, rund 40 % gingen sozialversicherungsfreien Beschäftigungen wie Zeitungsverkauf, Betteln oder Flaschensammeln nach. 108 Patient*innen gaben ihren Krankenversicherungsstatus an: 60 besaßen eine gültige Krankenversicherung. 79 Patient*innen machten Angaben über Haftaufenthalte in der Vergangenheit: 51 gaben an, mindestens einmal im Gefängnis gewesen zu sein.

### Ursachen, Auslöser und Risikofaktoren der Wohnungslosigkeit

Die Gründe der Wohnungslosigkeit, die von den Patient*innen angegeben wurden, ließen sich in 4 größere Themenkomplexe gliedern (Tab. 2). Als häufigste Ursache der Wohnungslosigkeit wurde die psychische Erkrankung genannt, gefolgt von Beziehungsabbrüchen, zu denen sowohl die Trennung von Mitbewohnenden (Lebenspartner*in oder Eltern) als auch deren Tod gezählt wurden. Bei einem Viertel waren materielle Gründe Ursache der Wohnungslosigkeit. Hierunter fielen sowohl Mietschulden als auch Wohnungsverlust infolge eines Arbeitsplatzverlustes. Circa 15 % gaben einen Haftantritt als Ursache der Wohnungslosigkeit an. Auch in der Vergangenheit waren Haftaufenthalte häufig. Charakterisiert ist die Studienpopulation zudem durch einen hohen Anteil belastender Lebensereignisse in Kindheit und Adoleszenz, die von den Autor*innen in den Biografien der Patient*innen identifiziert wurden.

**Table 2:** 

	Anzahl (%)
*Ursachen/Auslöser für Wohnungslosigkeit angegeben*	89 (79,5)
Eigene psychische Erkrankung	32 (36,0)
Beziehungsabbrüche	31 (34,8)
Materielle Gründe	22 (24,7)
Haftantritt	13 (14,6)
*Haftaufenthalte in der Vergangenheit, n* *=* *79*	51 (64,6)
*Belastende Lebensereignisse in Kindheit und Adoleszenz, n* *=* *65*	
Heimerziehung/früher Auszug aus dem Elternhaus	15 (23,1)
Psychische Erkrankung der Eltern/eines Elternteils	14 (21,5)
Sexueller Missbrauch	10 (15,4)
Gewalt in der Familie	8 (12,3)
Tod eines Elternteils/Trennung der Eltern	7 (10,1)
Flucht/Migration	1 (1,5)

### Verlauf von Betreuung im Gesundheitszentrum und Wohnungslosigkeit

Insgesamt dauerte die Betreuung der Patient*innen im Gesundheitszentrum im Median 2,0 Jahre (IQR = 4,0, Spannweite 2 Tage–12,7 Jahre). Bezüglich der Wohnsituation zum Ende der Betreuung zeigte sich, dass 106 Patient*innen weiter wohnungslos waren. 6 Patient*innen wohnten in einem mietvertraglich abgesicherten Wohnraum und konnten somit die Wohnungslosigkeit beenden (Tab. 3).

**Table 3:** 

	Anzahl (%)
*Betreuung im Gesundheitszentrum für Obdachlose, n* *=* *112*	
Davon > 1 Jahr	77 (68,7)
Davon > 5 Jahre	24 (21,4)
*Wohnsituation bei Ende der Betreuung im Gesundheitszentrum*	
Weiterhin wohnungslos	106 (94,6)
Wohnungslosigkeit beendet	6 (5,4)
*Gründe für Rückfall in die Obdachlosigkeit, n* *=* *51*	
Verstoß gegen die Hausordnung der Einrichtung der Wohnungslosenhilfe (Kündigung)	42 (25,9)
Unzufriedenheit mit Einrichtung der Wohnungslosenhilfe (freiwilliger Auszug)	22 (13,6)
Ordentliche Entlassung aus somatischer Klinik	27 (16,7)
Entlassung gegen ärztlichen Rat aus somatischer Klinik	21 (13,0)
Ordentliche Entlassung aus psychiatrischer Klinik	6 (3,7)
Entlassung gegen ärztlichen Rat aus psychiatrischer Klinik	4 (2,5)
Unzufriedenheit mit Suchtrehabilitation (freiwilliger Abbruch)	6 (3,7)
Nach Haftentlassung	21 (13,0)
Beziehungsabbruch (Auszug aus gemeinsamer Mietwohnung)	5 (3,1)
Sonstige Gründe	8 (5,0)

Es wurde des Weiteren analysiert, wie oft und aus welchem Grund die Patient*innen im Verlauf der Betreuung von einer besseren, d. h. gemäß ETHOS-Typologie nicht als „obdachlos“ definierten Wohnsituation (Kat. 3–13 oder Mietwohnung) ausgehend, wieder obdachlos (Kat. 1–2) wurden. Insgesamt wurde bei 102 Patient*innen (91,1 %) mindestens eine wiederholte Episode der Obdachlosigkeit dokumentiert, im Mittel 2,7 Episoden (Median 1, SD = 3,5, IQR = 2, Spannweite 1–28). Bei einer mittleren Betreuungsdauer von 35 Monaten bedeutet dies, dass im Schnitt ca. alle 13 Monate eine wiederholte Episode der Obdachlosigkeit beobachtet werden konnte.

### Zeitlicher Zusammenhang von psychischen Erkrankungen und Wohnungslosigkeit

Bei 106 Patient*innen (94,6 % der Studienpopulation) konnte der zeitliche Zusammenhang des Auftretens einer oder mehrerer psychischer Erkrankungen in Bezug auf den Beginn der Wohnungslosigkeit bestimmt werden. Bei 90 Patient*innen (84,9 %, 95 %-KI 77,4; 91,5) gingen eine oder mehrere psychische Erkrankungen dem Beginn der Wohnungslosigkeit voraus.

Bei 16 Patient*innen (15,1 %, 95 %-KI 8,5; 22,6) hingegen fanden sich keine Hinweise auf eine psychische Erkrankung vor Beginn der Wohnungslosigkeit, sie waren somit mutmaßlich zuerst wohnungslos und sind erst nach Beginn der Wohnungslosigkeit psychisch erkrankt.

Bei 46 Patient*innen nahm die Anzahl der gestellten Diagnosen während der Wohnungslosigkeit zu. Bei 54 Patient*innen blieb die Anzahl der Diagnosen gleich. Bei 6 Patient*innen verringerte sich die Anzahl der Diagnosen, in dieser Gruppe waren 5 drogenabhängige Patient*innen, die nach Beginn der Wohnungslosigkeit wieder clean wurden. Die Anzahl dokumentierter psychischer Erkrankungen für die gesamte Studienpopulation stieg im Laufe der Wohnungslosigkeit von durchschnittlich 1,58 psychischen Erkrankungen (95 %-KI 1,38; 1,78) vor Beginn der Wohnungslosigkeit um 25,5 % auf 2,12 (95 %-KI 1,95; 2,28) an.

**Figure 1:**
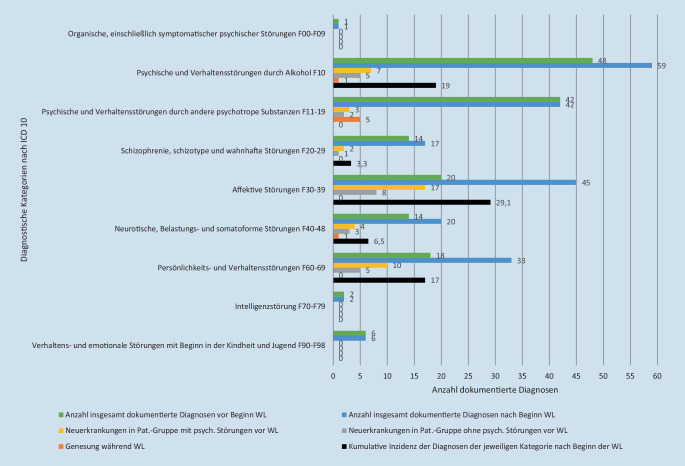

Die Diagnosegruppe affektive Störungen (ICD-10 F30-39) und die Diagnose Alkoholabhängigkeit (ICD-10 F10) zeigten die höchste kumulative Inzidenz in der Periode der Wohnungslosigkeit. Von den 86 zuvor nicht an affektiven Störungen leidenden Personen entwickelten 25 während der Wohnungslosigkeit eine affektive Störung (17 in der Patientengruppe mit psychischen Erkrankungen vor Beginn der Wohnungslosigkeit; 8 in der Patientengruppe ohne psychische Erkrankungen vor Beginn der Wohnungslosigkeit), dies ergibt eine kumulative Inzidenz von 29,1 % (Berechnung siehe Erläuterung zu Abb. 1). Bei den 20 bereits zuvor an dieser Diagnosegruppe erkrankten Personen ließen sich keine Hinweise auf eine Genesung finden. Von den 58 zuvor nicht alkoholabhängigen Personen entwickelten 11 eine Alkoholabhängigkeit (Kumulative Inzidenz 19 %), von den 48 zuvor alkoholabhängigen Personen konnte eine Person diese überwinden. Die entsprechenden Zahlen der restlichen Diagnosegruppen lassen sich Abb. 1 entnehmen.

### Mit frühem Beginn der Wohnungslosigkeit assoziierte Faktoren

Patient*innen ohne Schulabschluss waren zu Beginn ihrer Wohnungslosigkeit im Mittel 13,2 Jahre jünger als Patient*innen mit Abitur (95 %-KI −23,3; −3,4, *p* = 0,005); Patient*innen mit Hauptschulabschluss waren im Mittel 9,8 Jahre jünger (95 %-KI −18,0; −1,8, *p* = 0,012; Tab. 4). Patient*innen mit der Diagnosegruppe psychische und Verhaltensstörungen durch andere psychotrope Substanzen als Alkohol (ICD-10 F11–19) waren im Vergleich zu den Patient*innen anderer Diagnosegruppen im Mittel 6,7 Jahre jünger (95 %-KI −11,3; −2,1, *p* = 0,004). Tendenziell zeigten sich auch Haftaufenthalte in der Vergangenheit mit früher Wohnungslosigkeit assoziiert (*p* = 0,056).

**Table 4:** 

Variable	*n* (%)	Alter M (±SD)	*p*-Wert
*Geschlecht*			
Männlich	84 (80,0)	39,1 (12,0)	0,311 ^a^
Weiblich	21 (20,0)	42,1 (11,5)	–
*Belastende Lebensereignisse in Kindheit und Adoleszenz*			
Ja	40 (64,5)	37,9 (10,8)	0,191 ^a^
Nein	22 (35,5)	42,0 (13,0)	–
*Schulabschluss*			
Ohne Abschluss	13 (13,3)	33,3 (9,82)	*0,007*^*b*^
Hauptschulabschluss	34 (34,7)	36,7 (11,4)	–
Realschulabschluss	36 (36,7)	41,1 (12,3)	–
Abitur	15 (15,3)	46,6 (8,86)	–
*Haftaufenthalte in der Vergangenheit*			
Ja	49 (63,6)	36,5 (11,3)	0,056 ^a^
Nein	28 (36,4)	41,9 (13,0)	–
*Psychische Erkrankung vor Beginn der WL*			
Ja	85 (84,2)	39,8 (11,4)	0,664 ^a^
Nein	16 (15,8)	38,4 (14,3)	–
*Psychische und Verhaltensstörungen durch Alkohol (F10)*			
Ja	46 (42,9)	38,7 (10,2)	0,424 ^a^
Nein	59 (56,2)	40,5 (13,1)	–
*Psychische und Verhaltensstörungen durch andere psychotrope Substanzen als Alkohol (F11–19)*			
Ja	40 (38,1)	35,5 (11,5)	*0,004*^*a*^
Nein	65 (61,9)	42,3 (11,3)	–
*Schizophrenie, schizotype und wahnhafte Störungen (F20–29)*			
Ja	13 (12,4)	39,4 (10,2)	0,930 ^a^
Nein	92 (87,6)	39,7 (12,2)	–
*Affektive Störungen (F30–39)*			
Ja	19 (18,1)	43,1 (12,0)	0,169 ^a^
Nein	86 (82,9)	38,9 (11,8)	–
*Neurotische, Belastungs- und somatoforme Störungen (F40–49)*			
Ja	13 (12,4)	43,2 (11,9)	0,262 ^a^
Nein	92 (87,6)	39,2 (11,9)	*–*
*Persönlichkeits- und Verhaltensstörungen (F60–69)*			
Ja	16 (15,2)	40,3 (12,3)	0,839 ^a^
Nein	89 (84,8)	39,6 (11,9)	–

Signifikante Ergebnisse (*p* < 0,05) kursiv

*F* Diagnosegruppe für psychische und Verhaltensstörungen mit Untergruppen nach internationaler Klassifikation der Krankheiten (ICD-10), *M* Mittelwert, *SD* Standardabweichung, *WL* Wohnungslosigkeit

^a^zweiseitiger und ungerichteter t‑Test, ^b^ einfaktorielle ANOVA (Post-hoc-Test: Games-Howell)

### Mit langem Verlauf der Wohnungslosigkeit assoziierte Faktoren

Es zeigten sich die Variablen Haftaufenthalte in der Vergangenheit und psychische und Verhaltensstörungen durch Alkohol (ICD-10 F10) signifikant mit einer längeren Gesamtdauer der Wohnungslosigkeit assoziiert (Tab. 5). Patient*innen, die vor Erstkontakt im Gesundheitszentrum einen oder mehrere Haftaufenthalte verbracht hatten, waren durchschnittlich 27,8 Monate (ca. 2,3 Jahre) länger wohnungslos als Patient*innen ohne Hafterfahrung (95 %-KI 1,22; 54,3, *p* = 0,041). Patient*innen mit der Diagnose psychische und Verhaltensstörungen durch Alkohol vor oder während der Wohnungslosigkeit waren im Vergleich zu den anderen Patient*innen durchschnittlich 36,5 Monate länger wohnungslos (95 %-KI 14,7; 58,4, *p* = 0,001).

**Table 5:** 

Variable	*n* (%)	M (±SD)	*p*-Wert
*Geschlecht*			
Männlich	84 (80,0)	76,5 (59,3)	0,088 ^a^
Weiblich	21 (20,0)	52,0 (54,2)	–
*Haftaufenthalte in der Vergangenheit*			
Ja	49 (63,6)	76,8 (62,6)	*0,041*^*a*^
Nein	28 (36,4)	49,1 (42,9)	–
*Schulabschluss*			
Ohne Abschluss	13 (13,3)	67,0 (39,3)	0,208 ^b^
Hauptschulabschluss	34 (34,7)	82,4 (66,9)	–
Realschulabschluss	36 (36,7)	70,7 (55,9)	–
Abitur	15 (15,3)	44,6 (45,6)	–
*Psychische Erkrankung vor Beginn der WL*			
Ja	85 (84,2)	69,6 (58,4)	0,323 ^a^
Nein	16 (15,8)	85,8 (66,7)	–
*Anzahl psychischer Erkrankungen nach Beginn der WL*			
Eine	25 (23,8)	64,1 (61,6)	0,451 ^a^
Mehr als eine	80 (76,2)	74,5 (58,3)	–
*Psychische und Verhaltensstörungen durch Alkohol (F10)*			
Ja	57 (54,3)	88,3 (64,5)	*0,001*^*a*^
Nein	48 (45,7)	51,8 (44,4)	–
*Psychische und Verhaltensstörungen durch andere psychotrope Substanzen als Alkohol (F11–19)*			
Ja	50 (47,6)	62,8 (52,1)	0,142 ^a^
Nein	55 (52,4)	79,7 (63,8)	–
*Schizophrenie, schizotype und wahnhafte Störungen (F20–29)*			
Ja	17 (16,2)	60,7 (43,4)	0,406 ^a^
Nein	88 (83,49)	73,7 (61,4)	–
*Affektive Störungen (F30–39)*			
Ja	42 (0,40)	73,6 (56,6)	0,778 ^a^
Nein	63 (0,60)	70,3 (60,8)	–
*Neurotische, Belastungs- und somatoforme Störungen (F40–49)*			
Ja	19 (18,1)	66,4 (60,2)	0,685 ^a^
Nein	86 (81,9)	72,7 (58,9)	–
*Persönlichkeits- und Verhaltensstörungen (F60–69)*			
Ja	31 (29,5)	64,3 (58,8)	0,411 ^a^
Nein	74 (70,5)	74,7 (59,0)	–

*n* = 105, M = 71,6 Monate, SD = 58,9, Spannweite 2 Wochen–255,9 Monate

Signifikante Ergebnisse (*p* < 0,05) kursiv

*M* Mittelwert, *SD* Standardabweichung, *WL* Wohnungslosigkeit

^a^zweiseitiger und ungerichteter t‑Test

^b^einfaktorielle ANOVA (Post-hoc-Test: Games-Howell)

Die Anzahl wiederholter Episoden der Obdachlosigkeit war signifikant mit der Gesamtdauer der Wohnungslosigkeit assoziiert. Durchschnittlich verlängerte sich die Gesamtdauer pro Episode um 7,9 Monate (lineare Regression, *p* < 0,001).

## Diskussion

Probleme wie Wohnungslosigkeit müssen als multikausal betrachtet werden, und „eine Interdependenz zwischen Wohnungslosigkeit, Arbeitslosigkeit, psychischen Belastungen und erhöhten Erkrankungsrisiken“ [8] wird in der Wissenschaft angenommen. Die vorliegenden Ergebnisse zeigen, dass die große Mehrheit der untersuchten Betroffenen bereits vor Eintritt der Wohnungslosigkeit psychisch erkrankt war. Circa ein Drittel der Studienpopulation gaben dabei die psychische Erkrankung als direkte Ursache der Wohnungslosigkeit an, sodass hier eine zugrunde liegende Kausalität angenommen werden kann. Ergebnisse internationaler Studien [20–23] sowie deutscher Forschung [4, 9, 24, 25] werden hiermit bestätigt. Auch in der SEEWOLF-Studie wurde bei 85 % der Teilnehmer*innen eine psychische Störung vor Beginn der Wohnungslosigkeit identifiziert [9]. Während dort jedoch eine Überschätzung durch einen Selektionsbias diskutiert wurde, wurden die Daten unserer Analyse in einem für alle wohnungslosen Menschen zugänglichen Versorgungssetting erhoben. Gezeigt werden konnte auch, dass Wohnungslosigkeit zu einer Verschlechterung des psychischen Gesundheitszustandes führt, sodass eine aktuell unzureichende psychiatrische Versorgung dieser vulnerablen Gruppe konzediert werden kann. Die Erkenntnis, dass ein Großteil der psychischen Erkrankungen wohnungsloser Personen bereits vor Eintreten der Wohnungslosigkeit beginnt, spricht besonders für die Notwendigkeit einer verbesserten Kooperation zwischen den sozialpsychiatrischen Diensten und der Wohnungsnotfallhilfe sowie vermehrter aufsuchender Arbeit und Kontaktaufnahme mit Gruppen, die von Wohnungslosigkeit bedroht sind.

Die Multikausalität von Wohnungslosigkeit wurde durch eine Identifizierung von Einflussfaktoren für einen frühen Beginn der Wohnungslosigkeit weiter exploriert. Es konnte eine Subgruppe der Studienpopulation charakterisiert werden, die vergleichsweise jung wohnungslos wurde und damit inhaltlich den* Youth Homeless* in der englischsprachigen Literatur entspricht [22, 26]. Auch in Deutschland scheint es damit eine Untergruppe wohnungsloser junger Menschen zu geben, für die insbesondere ein früher Ausstieg aus der Schulbildung und eine Drogenabhängigkeit ineinandergreifende Schlüsselfaktoren in der Entstehung von Wohnungslosigkeit sind [27]. Unterstützt wird diese These durch den Befund unserer Studie, dass die kumulative Inzidenz der Drogenabhängigkeit in der Periode der Wohnungslosigkeit im Vergleich zu den anderen relevanten psychischen Erkrankungen vernachlässigbar ist. Die Drogenabhängigkeit entstand demnach hauptsächlich vor Beginn der Wohnungslosigkeit. In der englischsprachigen Literatur wurden die Wege verschiedener Subgruppen in die Wohnungslosigkeit bereits intensiv beforscht, um von Wohnungslosigkeit bedrohte Gruppen identifizieren zu können [26, 28, 29]. In Deutschland liegen vergleichbare Studien nicht vor, weitere derartige Subgruppenanalysen wären in Anbetracht der zunehmenden Diversität und Komplexität der Gruppe der wohnungslosen Personen sicher wertvoll.

Ein weiterer Aspekt der Entstehung von Wohnungslosigkeit, der in der deutschen Forschung bislang wenig Berücksichtigung fand, ist der Zusammenhang mit unbewältigten, belastenden Lebensereignissen in der Kindheit und Jugend. Die bestehende Literatur zählt diese zu den beständigsten Prädiktoren für Wohnungslosigkeit [11–13]. In der vorliegenden Studie wurde versucht, in den Biografien der Patient*innen entsprechende Lebensereignisse, die anhand der vorhandenen Literatur definiert wurden, zu explorieren. Da die Mehrheit der Studienpopulation ein oder mehrere entsprechende belastende Lebensereignisse erwähnte, kann eine zugrunde liegende Interdependenz angenommen werden. Genauere Analysen belastender Lebensereignisse unter wohnungslosen Menschen, auch qualitativer Art, könnten von großem Nutzen für ein besseres Verständnis der hier aufgrund der eingeschränkten Datenqualität nur rudimentär untersuchten Zusammenhänge sein.

Um Strategien zur Verhinderung von Wohnungslosigkeit entwickeln zu können, kann neben der Identifizierung verschiedener Wege in die Wohnungslosigkeit die wissenschaftliche Analyse von Langzeitwohnungslosigkeit ein vielversprechender Ansatz sein. Es konnte ein signifikanter Zusammenhang von Haftaufenthalten mit Langzeitwohnungslosigkeit identifiziert werden, der im englischsprachigen Raum bereits gut dokumentiert ist. So scheinen wohnungslose Personen ein höheres Risiko zu besitzen, inhaftiert zu werden [14, 30] und auch länger inhaftiert zu sein, als vergleichbare, vor dem Haftaufenthalt nicht wohnungslose Personen [15, 16]. Gründe hierfür könnten unter anderem in der zunehmenden, durch die Lebensumstände der Wohnungslosigkeit bedingten Kriminalisierung liegen, die zu vorübergehenden Inhaftierungen führen könnte [31]. In diesem Zusammenhang erscheint eine Abschaffung der sozial fragwürdigen Ersatzfreiheitsstrafen nach §43 Strafgesetzbuch (StGB) für diese Gruppe, zumindest bei kleineren Delikten wie dem Fahren im öffentlichen Personennahverkehr ohne Fahrschein, ein probates Mittel zur Entkriminalisierung zu sein. Die kurzfristige Abschaffung in Berlin im Zuge der COVID-19-Pandemie hat bereits gezeigt, dass dies politisch einfach umzusetzen wäre [32]. Umgekehrt scheinen Haftaufenthalte auch Langzeitwohnungslosigkeit zu fördern. Nach der Entlassung können Barrieren an der Schnittstelle zwischen den Sozialdiensten der Haftanstalten und den Sozialämtern [33] zu ungesicherten Entlassungen auf die Straße und zu einer signifikanten Verlängerung der Wohnungslosigkeit führen. Eine intensivere Auseinandersetzung der hiesigen Wohnungslosenforschung mit dieser Problematik scheint dringend angebracht, damit die Zusammenhänge zwischen Haftaufenthalten und Wohnungslosigkeit noch genauer untersucht werden.

Ein weiterer Zusammenhang besteht zwischen Langzeitwohnungslosigkeit und Alkoholabhängigkeit. Unsere Studie bestätigt eine Untersuchung aus München, in der alkoholabhängige wohnungslose Menschen signifikant länger wohnungslos waren als solche, die nicht alkoholabhängig waren [30]. Ein Erklärungsansatz für diese signifikante Verlängerung ist, dass alkoholkranke wohnungslose Menschen in eine Nische fallen, für die es überraschenderweise immer noch zu wenige spezialisierte therapeutische Einrichtungen gibt. Die Arbeitsgruppe in München beschrieb die Notwendigkeit solcher Einrichtungen bereits Ende der 1990er-Jahre [30], die vorliegenden Ergebnisse deuten darauf hin, dass diesbezüglich bis heute Verbesserungsbedarf besteht. Vielleicht wäre es an der Zeit, vorhandene Dogmen im Bereich der Suchtmedizin und auch Wohnungslosenhilfe zu hinterfragen, die zu oft an einer Abstinenz von Alkohol orientiert sind, die nicht zeitgemäß und für die Betroffenen nicht erreichbar ist [34, 35].

Die Versorgung mit Wohnraum für wohnungslose Personen beruht in Deutschland auf einem abgestuften System von Hilfen, das Schritt für Schritt durchlaufen werden muss, bevor final eine Mietwohnung bezogen werden darf [36]. Wiederholte Abstürze aus diesem Hilfesystem können zu einer langfristigen Verstetigung der Wohnungslosigkeit führen, die bereits als „Folge der Auseinandersetzung Wohnungsloser mit dem Hilfeangebot und seiner Mangelhaftigkeit“ [37] zusammengefasst wurde. Im Gegensatz zum englischsprachigen Raum, in dem diese Langzeitwohnungslosigkeit in Form der *Chronic *und *Episodic Homelessness *wissenschaftlich klar definiert ist und beforscht wird, gibt es hierzu in Deutschland aktuell nur vereinzelt empirische Untersuchungen [30]. In der vorliegenden Studie wurden deshalb Gründe von wiederholten Episoden der Obdachlosigkeit analysiert. Die Ergebnisse zeigen, welche Auswirkungen diese wiederholten Episoden in die Obdachlosigkeit auf die Dauer der Wohnungslosigkeit haben und welches Potenzial ein Verhindern dieser Episoden in der Verhinderung von Langzeitwohnungslosigkeit birgt. Die dargestellten Gründe, insbesondere die qualitativ unzureichenden und für psychisch erkrankte Personen nicht bedarfsgerechten Wohneinrichtungen mit kritikwürdigen Verhaltensregeln wie Abstinenzorientierung sowie Schnittstellenprobleme nach Aufenthalt in institutionellen Einrichtungen wie Krankenhäuser und Haftanstalten, scheinen von großer Bedeutung für die Chronifizierung von Wohnungslosigkeit zu sein.

### Limitationen

Durch den Rekrutierungsort der Studie im Gesundheitszentrum für Obdachlose in Berlin, das als Hybrid aus medizinischer Praxis, Sozialberatung, Suppenküche, Kleiderkammer und Tagestreffpunkt bis auf den Übernachtungssektor alle klassischen Rekrutierungsorte der Wohnungslosenforschung vereint, konnte eine große Stichprobenreichweite mit einer vielfältigen Wohnungslosenpopulation erreicht werden. Dennoch konnten aufgrund der fehlenden Dolmetscher im Gesundheitszentrum nur Daten von Patient*innen eingeschlossen werden, die über ausreichende Deutschkenntnisse für die ausführlichen Anamnesegespräche verfügten. Dies führte zu einer Verringerung der Repräsentativität der Studienpopulation bezüglich der Nationalitäten beziehungswiese der kulturellen Hintergründe. Die Aussagen können deshalb nur mit großer Vorsicht auf nichtdeutsche wohnungslose Menschen übertragen werden.

Eine repräsentative Stichprobe ist aufgrund der sehr heterogenen Gruppe der wohnungslosen Menschen schwierig zu ziehen, eine Totalerhebung logistisch und finanziell kaum realisierbar. Über den jeweiligen Ort der Rekrutierung der Stichprobe kommt es unvermeidlich zu Selektionseffekten. Diese Limitation ist seit Langem in der Forschung mit wohnungslosen Menschen bekannt [38]. Nach Vergleich mit Ergebnissen anderer Studien [1, 6, 9] ist die Stichprobe in den demografischen Merkmalen mit Ausnahme oben bereits genannter Limitation zumindest generalisierbar auf die Grundgesamtheit der wohnungslosen Menschen in Deutschland. Da die Gesundheits- und Sozialversorgung obdachloser Menschen in Berlin überwiegend durch spendenfinanzierte gemeinnützige Einrichtungen getragen wird, wie auch im an dieser Studie beteiligten Gesundheitszentrum, sind die Ressourcen für eine umfassende und standardisierte Dokumentation der Fälle zudem limitiert.

Die Stichprobe war nicht groß genug für eine weitergehende multivariate statistische Analyse. So war z. B. eine Adjustierung der Anzahl und Dauer wohnungsloser Episoden und Anzahl psychiatrischer Erkrankungen für die Variable Alter nicht möglich. Hierfür wäre erforderlich, dass die versorgenden Einrichtungen der Region auf Basis einer gemeinsamen standardisierten Dokumentation einen multizentrischen Datenzugang ermöglichen.

Die Entscheidung für eine Sekundärdatenanalyse von Patient*innenakten brachte mehrere Limitationen mit sich: Das Studiendesign erlaubte keine nichtwohnungslose Kontrollgruppe, Kausalitäten konnten so nicht bewiesen werden. Auf Art und Qualität der bereits vorliegenden Daten konnte kein Einfluss genommen werden: Es kann angenommen werden, dass in den Patient*innenakten mitunter potenziell wichtige Informationen fehlten, wie etwa bereits diagnostizierte, aber von den Patient*innen nicht erwähnte psychische Erkrankungen. Die interne Validität der Aussagen psychisch erkrankter Patient*innen, die bei der Anamnese etwa akut psychotisch oder akut intoxikiert gewesen sein können, muss zudem kritisch betrachtet werden. Insbesondere die Art der Diagnosestellung der psychischen Erkrankungen ist essenziell für die Interpretation der wichtigsten Ergebnisse dieser Studie, diese war aufgrund des Studiendesigns nicht standardisiert möglich. Je nach Untersucher*in unterschied sich die Diagnosestellung in ihrer Sensitivität: In die Studie eingeflossen sind etwa über Arztbriefe Diagnosen aus einem klinisch-stationären Setting, Verdachtsdiagnosen der langjährig erfahrenen Psychologin nach klinischem Interview sowie Verdachtsdiagnosen von nicht psychiatrisch geschultem medizinischen Personal. Diese Aspekte schmälern die Validität der Ergebnisse, insbesondere in Bezug auf die erste Fragestellung zum zeitlichen Zusammenhang zwischen psychischer Erkrankung und Wohnungslosigkeit und zur Veränderung von Anzahl und Schwere der psychischen Erkrankungen. Aufgrund der oft unsicheren Diagnosen wurde konsequenterweise nur eine grobe Einteilung der psychiatrischen Erkrankungen in ICD-10-Gruppen durchgeführt; eine kleinteiligere Unterscheidung, zum Beispiel zwischen den einzelnen affektiven Störungen oder zwischen den einzelnen Drogenabhängigkeitserkrankungen, war aufgrund der dargelegten Limitation nicht sinnvoll. Die Studie muss daher auch diesbezüglich in ihren Aussagen auf einer explorativen Ebene bleiben.

## Schlussfolgerung

Die weiterhin bestehende Problemlage der psychisch erkrankten wohnungslosen Menschen stellt eine komplexe Herausforderung für Betroffene, im Hilfssystem tätige Personen sowie für Politik und Wissenschaft dar. Die gegenwärtigen Antworten auf die Unterversorgung dieser vulnerablen Gruppe, die in dieser Studie aufgezeigt werden konnte, reichen dabei weder qualitativ noch quantitativ aus. Die psychosoziale Versorgung von psychisch Erkrankten muss explizit Strategien zur Prävention von Wohnungslosigkeit beinhalten. In der Wissenschaft sind dafür innovative Forschungsansätze notwendig, die die zahlreichen Limitationen in der Wohnungslosenforschung überwinden können. Es wird sich zeigen, ob die kommenden Strategien der neuen Bundesregierung, wie die geplante Integration des *Housing-first*-Konzepts^1^ [36], zur Verbesserung der Versorgung beitragen werden. Gewiss scheint, dass das ehrgeizige Ziel der Europäischen Union, die unfreiwillige Obdachlosigkeit bis 2030 zu beenden [39], nur durch enge Zusammenarbeit aller Akteure bewältigt werden kann.

## Footnotes

1 Als „housing first“ wird eine staatlich finanzierte, voraussetzungsfreie, unbefristete und mietvertraglich abgesicherte Unterbringung in einer Wohnung bezeichnet, die von verschiedenen Hilfestellungen und Beratungsangeboten begleitet wird.
